# Editorial: Combating cancer with natural products: Non-coding RNA and RNA modification

**DOI:** 10.3389/fphar.2023.1149777

**Published:** 2023-02-10

**Authors:** Yongye Huang, Yue Hou, Peng Qu, Yun Dai

**Affiliations:** ^1^ Key Laboratory of Bioresource Research and Development of Liaoning Province, College of Life and Health Sciences, Northeastern University, Shenyang, China; ^2^ National Cancer Institute, Frederick, MD, United States; ^3^ Laboratory of Cancer Precision Medicine, The First Hospital of Jilin University, Changchun, China

**Keywords:** natural products, cancer, non-coding RNAs, RNA methylation, circRNA

Cancer is a type of tumor with the ability of malignant proliferation, whereby cancer cells survive and multiply gradually out of the body’s control. The hallmarks of cancer have been clearly and comprehensively summarized by the Hanahan study group. The hallmarks of cancer have been described as sustaining proliferative signaling, evading growth suppressors, resisting cell death, enabling replicative immortality, inducing/accessing vasculature, activating invasion and metastasis, reprogramming cellular metabolism, avoiding immune destruction, deregulating cellular metabolism and avoiding immune destruction (Hanahan, 2022). Meanwhile, Hanahan (2022) notes that unravelling phenotypic plasticity, non-mutational epigenetic reprogramming, polymorphic microbiomes and senescent cells are likely to be incorporated into the cancer conceptualization of hallmarks. On this basis, the researchers have further revealed that cancer epigenetics, genomic instability and mutations play an important role in the acquisition of cancer hallmarks by cells (Ravi et al., 2022; Zhang et al., 2022).

The emergence of the concept of cancer epigenetics has inspired a wide range of researchers to conduct more in-depth research into cancer mechanisms and cancer treatments. It is generally assumed that epigenetic abnormalities are caused by interactions between multiple protein complexes and their components, such as histone modifications, DNA methylation mechanisms, chromatin remodeling proteins and polycomb (PcG) proteins (Tsai and Baylin, 2011). Eighty per cent of human genome transcripts are non-coding RNAs (ncRNAs) that can regulate gene expression to modulate cancer progression, stemness, migration, and metastasis. According to the localization, length and function, ncRNAs can be classified into several types: rRNA, tRNA, snRNA, snoRNA, siRNA, piRNA, microRNA, lncRNA, and circRNA. Among them, microRNA, lncRNA, and circRNA play a critical role in tumorigenesis and cancer therapy. Alternative splicing defects are often found in cancer metastasis, invasion and the generation of drug resistance.

The crosstalk among lncRNA, microRNA, and circRNA has become a critical regulatory mechanism in tumorigenesis and cancer progression. Long non-coding RNAs are RNA molecules with transcriptional lengths longer than 200 nt and lacking protein-coding capabilities, which regulate gene expression by interacting with DNA, RNA and proteins (Xing et al., 2021). Epigenetic and non-coding RNA abnormalities can lead to uncontrolled gene expression, including epigenetic silencing of tumor suppressor microRNAs, epigenetic aberrations caused by microRNAs, epigenetic activation of oncogenic microRNAs and abnormalities in natural compounds that regulate microRNA expression by epigenetic mechanisms (Farooqi et al., 2019). CircRNA is an endogenous RNA with a covalent closed loop that plays an important part in the epigenetic regulation of transcriptional and post-transcriptional genes, and its dysregulation is associated with tumorigenesis and metastasis (Huang and Zhu, 2021). As research intensified, lncRNA/circRNA has been described as a sponge for microRNA in the competitive endogenous RNA (ceRNA) regulatory model, which proceeds to regulate the downstream target genes of microRNA (Han et al., 2020). In a ceRNA regulatory network research on circRNA-lncRNA-miRNA-mRNA of Acute myeloid leukemia, it was demonstrated that circRNA and lncRNA participate in the complex post-transcriptional regulation of the ceRNA network (Cheng et al., 2020). The lncRNAs, circRNAs, microRNAs and snRNAs among ncRNAs have been shown to affect cancer progression through the alternative splicing process, thereby regulating the alternative splicing process and generating alternatively spliced isoforms (Liu et al., 2021). Therefore, investigation into the transcriptional regulatory functions of non-coding RNAs is likely to contribute to finding appropriate strategy for cancer therapy.

Natural products have been used in cancer treatment for a long time and some recent mechanistic studies have attracted much attention (Xiang et al., 2019). The research on natural products to combat tumors is abundant, and their functions in regulating the tumor microenvironment and activating the body’s immune response to kill cancer cells have been proven in various aspects. Natural medicines have vital regulatory functions at the genetic, epigenetic and signalling pathway transductions (Xiang et al., 2019; Huang et al., 2021; Zhang et al., 2022). The PI3K-Akt-mTOR pathway is one of the critical signaling pathways that may be targeted to inhibt cancer progression; afrocyclamin A, oridonin, salidroside, vitexin, arctigenin, cryptotanshinone, apigenin, and curcumin, are proposed to interfere with this pathway (Tewari et al., 2022). Natural products play a significant part in modulating the immune response against cancer. For example, saponins and flavonoids are two major groups of natural products that have shown excellent efficacy in reversing the tumor immunosuppressive microenvironment in conjunction with cancer immunotherapy (Tewari et al., 2022). Natural products have been shown to selectively trigger an effective host immune reaction against cancer cells, which strongly supports the strategy of turning “cold” tumors into “hot” ones to increase the efficiency of immune checkpoint inhibitor responses for cancer therapy (Atanasov et al., 2021). Natural products such as curcumin, resveratrol, apigenin, quercetin, berberine, genistein, epigallocatechin gallate, and parthenolide play a part in regulating epigenetic abnormalities (Huang et al., 2018; Xiang et al., 2019; Li et al., 2022; Zhang et al., 2022). Increasingly clear knowledge of the epigenetic mechanisms in cancer development has led to a search for more accurate and efficient anticancer agents.

**FIGURE 1:**
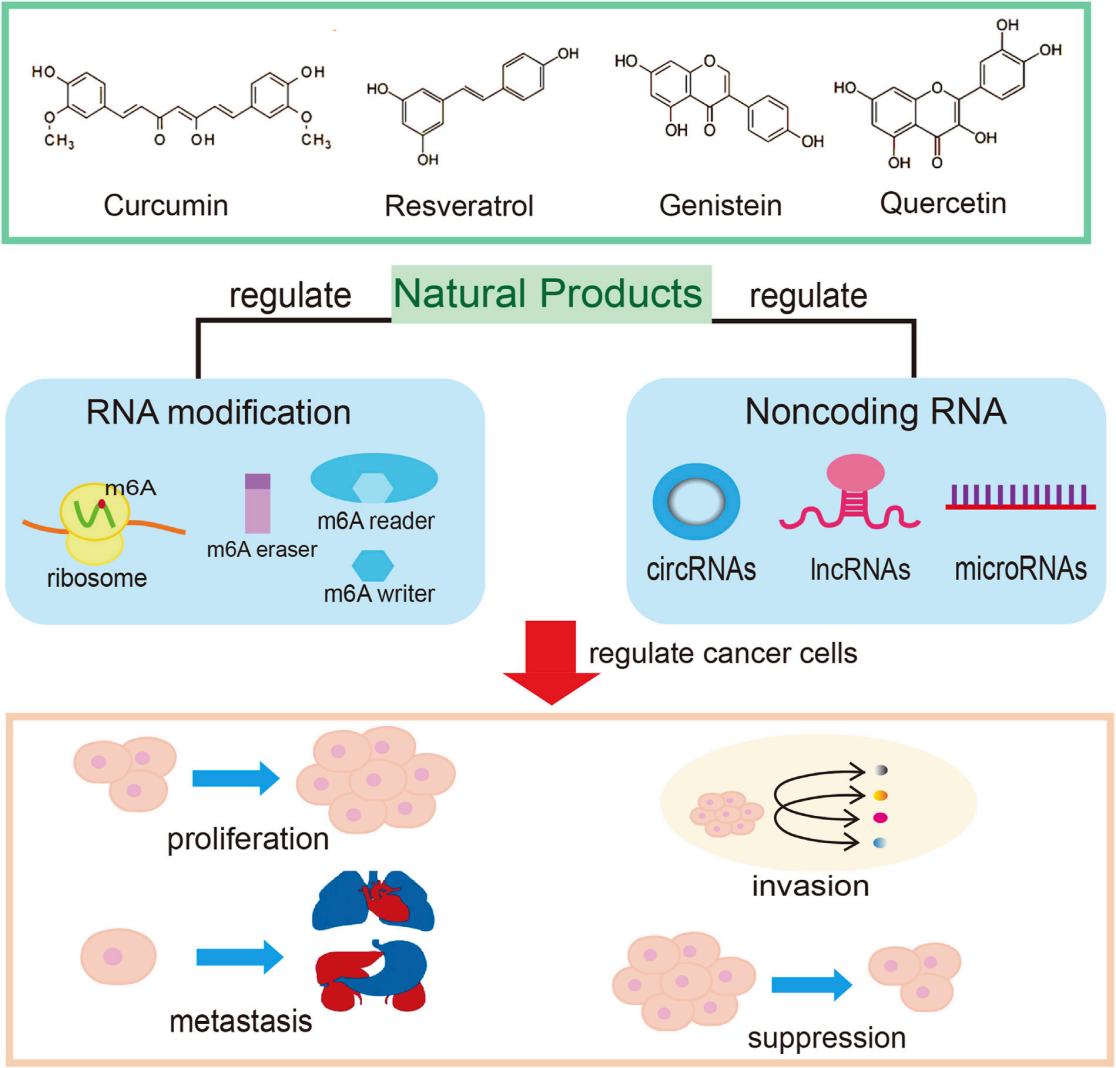
ncRNAs and RNA modifications underlying natural products-based cancer treatment.

Through continuous attempts to dismantle the threat of epigenetic abnormalities from environmental and dietary factors using natural products, many viable therapeutic options are available for cancer prevention and anti-cancer drug development (Figure 1). However, even though some natural products are currently undergoing clinical evaluation for cancer treatment, a large proportion of natural products are far from being used in a clinical setting. As previously mentioned, ncRNAs and RNA modifications are critically important in cancer development and therapy. Targeting these actors may actually contribute to the anti-tumor effects of natural products through inhibition of proliferation, invasion and metastasis (Homayoonfal et al., 2021). In addition, natural products such as paclitaxel, curcumin, resveratrol and genistein exert their antiproliferative and/or proapoptotic effects by modulating one or more miRNAs, thereby inhibiting cancer cell growth, inducing apoptosis and enhancing the effects of conventional cancer therapy (Xiang et al., 2019; Zhang et al., 2020). Therefore, the prospect of using the regulatory function of natural products on ncRNA to treat cancer is quite encouraging.

Numerous investigations have confirmed that RNA modification has emerged as a primary mechanism in controlling cell transcriptome and proteome during cancer development (Xiang et al., 2019; Zhang et al., 2022). More than one hundred RNA modifications, including mRNA cap modifications, N6-methyladenosine (m6A), and RNA editing. Among them, m6A modification is the most abundant RNA modification and exerts function in various biological processes in eukaryotes. Increasing evidence has confirmed that m6A modification could regulate the expression and function of ncRNAs, subsequently modulating the consequence of tumorigenesis and cancer therapy. The m6A modification is closely associated with cancer metastasis, stemness, drug resistance and microenvironment remodeling. Previously, investigators have suggested that m6A modification is likely to be one of the upstream regulatory mechanisms for lncRNAs differentially expressed in tumors. However, the clinical application of natural products that target RNA modifications for cancer treatment is rare, and research related to the regulation of m6A modifications by natural products is also infrequent. In a natural product screening study of m6A modulators, it was noted that using artificial intelligence-assisted technology combined with database analysis of traditional drugs and natural products holds promise for the development of more effective m6A modification-mediated therapeutic agents to inhibit tumor progression (Deng et al., 2022). The mechanism linking natural drugs and RNA modification should be thoroughly explored by taking full advantage of artificial intelligence, gene editing, RNA interference and other technologies in the future studies. Screening safe and efficient natural drugs as RNA modification modulators will bring new hope for cancer clinical treatment.
